# Institutions for the Insane in Prussia, Austria, and Germany
*Extracted from No. 54 of "The American Journal of the Medical Science," edited by Dr. Isaac Hay. "Institutions for the Insane in Prussia, Austria, and Germany." By Pliny Earle, M.D., one of the Visiting Physicians to the Lunatic Asylum of the City of New York, &c. Utica, 1853. 8vo, pp. 229.


**Published:** 1854-07-01

**Authors:** 


### INSTITUTIONS FOR THE INSANE IN PRUSSIA, AUSTRIA, AND GERMANY.*

This volume contains an interesting record of a very extensive personal examination of many of the numerous institutions for the insane in Prussia, Austria, and Germany.

Familiar as we have become with all the prominent hospitals for the treatment of mental disease in Great Britain and France, only a limited number beyond these countries have been seen by any of our professional men, who have visited Europe for the purpose of profiting by the improvements which, within the last twenty or thirty years, have been introduced into most of these institutions. "A general impression appeared to prevail, indicated, it is true, more by negative than positive signs, that, aside from the countries mentioned, the nations of Europe had made but little progress in this department of the profession, and hence could furnish us nothing commensurate with the labour and expense necessary to its acquisition," and yet the literature of the Germans on this branch of medicine is able and voluminous. Much of it is, perhaps, unprofitable, as being devoted to a zealous advocacy of specious theories, but still containing a great amount of valuable information, and exhibiting very strikingly the talent and industry which, in that region, are devoted to the study of mental diseases.

Various institutions for the care of the insane, too, are to be found throughout these countries, which have a deservedly high character for their liberal arrangements, and the admirable manner in which their whole service is performed.

During the summer of 1849, Dr. Earle visited many of these institutions, under peculiarly favourable circumstances for obtaining a knowledge of their actual condition. Long devoted to the study of diseases of the mind, and for several years engaged in the superintendence of a large American hospital, he went abroad with a degree of practical knowledge of the subject, and a familiarity with the wants of such establishments, which rendered him well qualified to judge of the excellences, as well as the defects, which are to be found in abundance in the different German institutions.

The first chapter of the work before us is devoted to a brief history of insanity in Germany, of the German periodical and other literature on the subject, and an interesting notice of the prominent men who have been distinguished in this speciality—which, although receiving only a brief notice at our hands, will well repay an attentive perusal.

The following estimate of German hospitals, as compared with our own, is interesting:—

"A large proportion of the buildings occupied as hospitals or asylums for the insane in Germany, were formerly monastic establishments. Their architectural arrangements are not only of a former age, but were adapted to a different purpose, and hence are less convenient than most of our institutions. Still, their conversion into asylums for the insane has already been productive of at least one advantage. It has accustomed the officers of these institutions to large

* Extracted from No. 54 of "The American Journal of the Medical Science," edited by Dr. Isaac Hay. "Institutions for the Insane in Prussia, Austria, and Germany." By Pliny Earle, M.D., one of the Visiting Physicians to the Lunatic Asylum of the City of New York, &c. Utica, 1853. 8vo, pp. 229.

rooms, so that, in the construction of new buildings, the principle of providing accommodations for the greatest number of patients in the least possible space does not enter into consideration. It is really a delightful treat to see the large, well-lighted, and airy corridors of Eichburg and the asylum at Halle. The number of cubic feet of inclosed space in the principal German institutions is probably not less than twice as great, in proportion to the number of patients, as those in the United States. Such asylums as have been recently erected, and specially designed for the purpose—as, for example, those of Halle, Illenau, and Eichburg—are great improvements upon the others, and yet, in point of convenience, are unequal to some of ours. In their asylums generally, the apartments for patients have not that finished aspect of comfort which is found in many of the American institutions. This is particularly owing to the universal absence of carpets. Yet, relatively to the prevailing customs of the people, they are probably as well furnished as ours. In the conveniences of the kitchen, the laundry, and the means of distributing food throughout the house, they are inferior. Cooking is rarely done by steam. I saw no wringing-press, and no dumb-waiter. Mechanical appliances for the purpose of bodily restraint are probably somewhat more extensively used than upon this side of the Atlantic."

It appears that, during this visit, Dr. Earle found several establishments in which he was shown through only a portion of the wards, and occasionally he had no opportunity of seeing those for violent patients. We trust few, if any, American superintendents can be found but that will cordially agree with Dr. Earle, that when a professional brother, engaged in the same speciality, visits an establishment for the purpose of becoming familiar with its arrangements, it is a duty, and ought to be a pleasure, to conduct him through every ward, and to throw open every part for his inspection. The credit should be given such a visitor that he comes "to learn the advantages of the institution, not to seek for demerits or matters for cavil."

In regard to moral treatment, Dr. E. considers the German asylums fully equal to those of the United States. "In the most important point of all—if reference be had to curative treatment, or the quietude, order, and hygienic condition of the patients—that of manual employment for the inmates, they are superior. The radical source of this superiority lies, undoubtedly, not in the more ardent wishes, or the greater efforts, of their superintendents for the welfare of their patients—for, in these respects, none can excel the officers of the American asylums—but in the education of the people, and the nature of the political governments under which they live. Obedience to authority becomes, by education, more a matter of principle or of habit. Furthermore, the asylums are more independent than ours, and the retention and management of patients more optional with the officers."

Of the forty-nine public, and eight private, establishments of which mention is made, and a more or less extended description given, in the volume before us, seventeen were visited by Dr. E. They embraced those of Sieberg, Andernach, Eberbach, Frankfort, Dusseldorf, Hildesheim, Halle, Berlin, Sonnenstein, Leubus, Brieg, Vienna, Hall, Giesing, Winnenthal, Illenau, and Stephansfield. Nine of these are among the thirteen which Dr. Julius calls the best in Germany.

Want of space prevents our giving a more extended notice of the author's visit, or referring to the many interesting facts and judicious criticisms scattered through the volume. We can heartily commend the work to the attention of all who take an interest in the insane, or are disposed to become familiar with the views of prominent German physicians on this important subject, and to learn from a competent observer the actual condition of the various institutions which, in that wide and populous region, are specially devoted to the treatment of the various forms of mental disease.

